# Insulin-Like Growth Factor 1 (IGF-1) Enhances the Protein Expression of CFTR

**DOI:** 10.1371/journal.pone.0059992

**Published:** 2013-03-28

**Authors:** Ha Won Lee, Jie Cheng, Olga Kovbasnjuk, Mark Donowitz, William B. Guggino

**Affiliations:** 1 Department of Physiology, Johns Hopkins University, School of Medicine, Baltimore, Maryland, United States of America; 2 Department of Medicine, Johns Hopkins University, School of Medicine, Baltimore, Maryland, United States of America; National Center for Scientific Research Demokritos, Greece

## Abstract

Low levels of insulin-like growth factor 1 (IGF-1) have been observed in the serum of cystic fibrosis (CF) patients. However, the effects of low serum IGF-1 on the cystic fibrosis transmembrane conductance regulator (CFTR), whose defective function is the primary cause of cystic fibrosis, have not been studied. Here, we show in human cells that IGF-1 increases the steady-state levels of mature wildtype CFTR in a CFTR-associated ligand (CAL)- and TC10-dependent manner; moreover, IGF-1 increases CFTR-mediated chloride transport. Using an acceptor photobleaching fluorescence resonance energy transfer (FRET) assay, we have confirmed the binding of CAL and CFTR in the Golgi. We also show that CAL overexpression inhibits forskolin-induced increases in the cell-surface expression of CFTR. We found that IGF-1 activates TC10, and active TC10 alters the functional association between CAL and CFTR. Furthermore, IGF-1 and active TC10 can reverse the CAL-mediated reduction in the cell-surface expression of CFTR. IGF-1 does not increase the expression of ΔF508 CFTR, whose processing is arrested in the ER. This finding is consistent with our observation that IGF-1 alters the functional interaction of CAL and CFTR in the Golgi. However, when ΔF508 CFTR is rescued with low temperature or the corrector VRT-325 and proceeds to the Golgi, IGF-1 can increase the expression of the rescued ΔF508 CFTR. Our data support a model indicating that CAL-CFTR binding in the Golgi inhibits CFTR trafficking to the cell surface, leading CFTR to the degradation pathway instead. IGF-1-activated TC10 changes the interaction of CFTR and CAL, allowing CFTR to progress to the plasma membrane. These findings offer a potential strategy using a combinational treatment of IGF-1 and correctors to increase the post-Golgi expression of CFTR in cystic fibrosis patients bearing the ΔF508 mutation.

## Introduction

Cystic fibrosis is a genetic disease caused by mutations in CFTR [1]. CFTR’s primary function is to move chloride ions across the plasma membrane of epithelial cells; this is a key function in the normal operation of several organs, including the airways, the intestinal tract, the pancreas, the epididymis, and the sweat duct (see [2] for a review). Dysfunctional chloride transport of mutant CFTRs leads to pathologically low levels of fluid, accompanied by altered composition in the airways, pancreatic duct, and intestinal tract, and it causes symptoms such as bacterial airway infections, chronic inflammation, growth retardation, male infertility, and obstruction of pancreatic ducts and the gastrointestinal tract. Failure to absorb fluid in the sweat ducts leads to high sweat chloride in patients, a symptom that has been used as a defining factor prior to the identification of the CF gene [3].

Because CFTR is localized at the cell surface to transport chloride ions, CFTR mutations resulting in improper localization (e.g., the most common mutation, ΔF508 CFTR) are particularly severe [4], [5]. Therefore, the processes involved in the trafficking of both wildtype and ΔF508 CFTR have been studied extensively (see [6] for a review). It is now well known that CFTR trafficking to the cell surface is regulated by PDZ proteins (the Golgi reassembly stacking protein [GRASP], CFTR-associated ligand [CAL], Na^+^/H^+^ exchanger regulatory factor [NHERF1/2], and CFTR-associated protein 70 [CAP70]), which bind to CFTR [7]–[10]. These proteins assemble CFTR into protein complexes in the ER, Golgi, or plasma membrane in polarized epithelial cells [8], [10] and ultimately regulate CFTR localization at the apical membrane by allowing CFTR to reach the plasma membrane, sequestering it within the cell, or targeting it for degradation (see [11] for review). For example, GRASP is localized to the Golgi [12]. When ER stress occurs, GRASP is phosphorylated and then binds to CFTR, leading to CFTR trafficking from the ER to the cell surface through a unique trafficking pathway [9]. CAL regulates the total and cell-surface expression of CFTR, either by enhancing the lysosomal degradation of CFTR [7] or allowing it to traffic to the plasma membrane, depending on which secondary proteins are bound to CAL [13]. At the plasma membrane, NHERF and CAP70 stabilize CFTR and allow CFTR to form an efficient functional complex [8], [14], [15].

As previously mentioned, CAL and its associated binding proteins regulate the lysosomal degradation and surface expression of CFTR [13], [16]. CAL is an adaptor protein that has multiple protein-interacting domains, including a PDZ domain that binds CFTR and two coiled-coil domains that are responsible for its Golgi localization. Syntaxin 6 (STX6), a soluble N-ethylmaleimide-sensitive factor-activating protein receptor protein (SNARE) protein, and TC10, a small GTPase of the Rho family, are known to bind to CAL in the region of the coiled-coil domains [17], [18].

STX6 is involved in intracellular vesicle trafficking [16], [18], [19]. Binding of STX6 to CAL reduces CFTR protein expression by increasing the transport of CFTR to the lysosome and thus promoting the lysosomal degradation of CFTR. Therefore, when bound to STX6, CAL is a negative regulator of both the total and cell surface expression of CFTR. In contrast, TC10 is a positive regulator of CFTR surface expression: By binding to CAL, it promotes the trafficking of CFTR to the plasma membrane and increases the total and cell-surface expression of CFTR [13]. Although the interaction of CFTR with CAL, TC10, and STX6 has been studied, how these pathways are regulated is not known. Information on how this regulation occurs certainly has implications for our understanding of both wildtype CFTR and the mutant ΔF508 CFTR.

Given that active TC10 promotes the trafficking of CFTR to the cell surface [13], the question to be asked is how TC10 is activated to increase the surface expression of CFTR.? IGF-1 and insulin activate TC10 through a GTP exchange factor in neurons and adipocytes [20], [21]. IGF-1 activates TC10 in neurons for neuronal growth, and insulin increases GLUT4 vesicle translocation in adipocytes for glucose uptake by activating TC10 [21]–[23]. Thus, it is likely that IGF-1 and/or insulin is(are) the upstream activator(s) of TC10 that ultimately regulate(s) CFTR protein expression. CF patients as well as CF pig models have low IGF-1 levels in their serum [24]–[27], an observation that also has implications for the pathophysiology of CF. Therefore, in this report, we tested whether IGF-1 alters CFTR expression. Dominant-negative TC10 was used to determine whether TC10 activation is required for any of the IGF-1-induced changes in CFTR protein expression. Our results showed that TC10, activated via IGF-1, does indeed alter the functional interaction between CFTR and CAL, ultimately increasing CFTR surface expression. We also suggest that IGF-1 may have therapeutic value in patients bearing the ΔF508 CFTR mutation.

## Materials and Methods

### Reagents and Antibodies

IGF-1, forskolin, and brefeldin A were obtained from Sigma. cDNAs encoding EYFP-tagged wildtype or mutant CFTR were subcloned with the pEYFP-C1 plasmid (Clontech), and cDNA encoding ECFP-tagged CAL was subcloned with the pECFP-C1 plasmid (Clontech), in the same manner that GFP was tagged to CFTR and CAL in previous studies [7], [28]. Anti-CFTR antibodies were obtained from the University of North Carolina (217) and Upstate (M3A7). Anti-GFP antibody (ab290) and anti-Na,K-ATPase antibody (464.6) were purchased from Abcam, anti-HA antibody (12CA5) from Roche, and anti-HA antibody conjugated with HRP from Sigma. Anti-GM130 antibody (35/GM130) was obtained from BD Bioscience and Alexa-647 goat anti-mouse IgG from Invitrogen.

### Cell Culture and Transfection

HeLa cells were cultured in Dulbecco’s modified Eagle’s medium (DMEM, Invitrogen) supplemented with 10% fetal bovine serum (FBS, Invitrogen) and penicillin/streptomycin (Invitrogen) in a humidified 37°C CO_2_ incubator. CFBE41o- cells (a cell line from bronchial epithelial cells of a cystic fibrosis patient [29], [30], kindly provided by Dr. Gruenert) were cultured in minimum essential medium Eagle (MEM, Invitrogen) with 10% FBS and penicillin/streptomycin, and CFBE-WTCFTR cells (CFBE41o- cells stably transfected with wildtype CFTR [30], kindly provided by Dr. Gruenert) were cultured in MEM with 10% FBS, penicillin/streptomycin and 300 µg/ml hygromycin B. For serum starvation, HeLa cells were cultured in DMEM with 0.5% FBS, and CFBE and CFBE-WTCFTR cells were cultured in MEM without FBS. Cells were transiently transfected with Lipofectamine 2000 reagent (Invitrogen) according to the manufacturer’s instruction and incubated for 24–48 hours.

### Coimmunoprecipitation and Western Blotting

Cells were washed twice with cold Dulbecco’s phosphate buffered saline (DPBS, Invitrogen), and then resuspended in lysis buffer (150 mM Tris, 50 mM NaCl, 1% Nonidet P-40, pH 7.4) with protease inhibitor cocktail (Roche). After 20 minutes on ice, the lysates were cleared by centrifugation at 15,000 g for 20 minutes. Pellets were discarded, and supernatants were used for further experiments. Protein G-Sepharose (GE Healthcare) was mixed with cleared lysates and primary antibodies and incubated on a rotary shaker at 4°C overnight. The beads were washed three times with lysis buffer and incubated in Laemmli buffer at 95°C for 5 minutes or at 42°C for 20 minutes. Total cleared lysates were denatured in Laemmli buffer at 42°C for 20 minutes. The protein concentration of each sample was measured with a BCA protein assay kit (Thermo Scientific), and equal amounts of total proteins were loaded on HCl-Tris gels.

Proteins were separated by SDS-PAGE and transferred to PVDF membranes (BioRad). After the transfer, the membranes were blocked with 5% nonfat milk in Tris-buffered saline with 0.05% Tween 20 (TBST) for 1 hour. The membranes were incubated with primary antibodies in 5% milk in TBST at 4°C overnight. The membranes were washed four times with TBST and incubated with Clean-Blot IP Detection Reagent (Thermo Scientific) or appropriate horseradish peroxidase-conjugated secondary antibodies (GE Healthcare).

### Cell-surface Biotinylation

After cells were washed four times with cold DPBS containing 1 mM Ca^2+^ and 1 mM Mg^2+^, they were incubated with 0.5 mg/ml sulfo-NHS-SS-biotin (Thermo Scientific) in DPBS with Ca^2+^ and Mg^2+^ at 4°C for 30 minutes. Free sulfo-NHS-SS-biotin was then quenched by washing with cold 192 mM glycine in DPBS four times. Cells were lysed with lysis buffer, and the lysates were centrifuged at 15,000 g at 4°C for 20 minutes. The supernatants were incubated with NeutrAvidin UltraLink Resin (Thermo Scientific) at 4°C for 5 hours or overnight. The beads were washed twice with lysis buffer, and proteins were eluted with Laemmli buffer at 42°C for 20 minutes. Biotinylated protein samples were analyzed by western blotting as described above.

### TC10 Activity Assays

HA-tagged TC10 [13] was transfected into cells. The transfected cells were serum-starved, and after 24 hours, the cells were treated with 0.1 µg/ml IGF-1 for another 24 hours. After a wash with cold DPBS, the cells were scraped in magnesium-containing lysis buffer (Millipore), and the cell lysates were incubated with GST-PAK-1 PBD-conjugated agarose beads (Millipore) for 1 hour and washed with the lysis buffer three times [31], [32]. Laemmli buffer was added to the beads and boiled for 5 minutes.

### Fluorescence Microscope and Acceptor Photobleaching Fluorescence Resonance Energy Transfer (FRET)

A Zeiss LSM 510 laser scanning system and 40x oil-immersion lens were used. Cells were seeded onto cover glasses or a glass-bottom dish. The following steps were done at room temperature: After a wash with cold DPBS, cells were fixed with 4% paraformaldehyde for 10 minutes. For immunostaining, after the fixation, cells were permeabilized with DPBS containing 0.1% Triton X-100 for 20 minutes and blocked with 1% bovine albumin serum or 5% goat serum (both from Sigma) for 1 hour. After blocking, the cells were incubated with primary antibodies and then with secondary antibodies conjugated to Alexa 647. Cells were mounted using ProLong Gold with DAPI (Invitrogen).

CFP was excited by a 458-nm Argon/2 laser, and the emission was detected with a 465–510 filter; YFP was excited by a 514-nm Argon/2 laser, and the emission was detected with a 520–555 filter. Alexa 647 was excited by a 633-nm HeNe633 and detected with a 646–700 filter. The 16-bit look-up table originally provided with ImageJ was applied to CFP images taken for FRET.

CFP/YFP pairs were used for acceptor photobleaching FRET. Images for CFP and YFP were collected before and after photobleaching with the 514-nm Argon/2 laser at the maximum intensity. From the images, the signal intensity of CFP was measured with ImageJ image software (NIH), and FRET efficiency was calculated using the following formula: 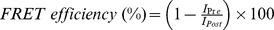, where 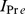 and 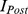 correspond to the CFP signal intensity (background signal was subtracted) before and after photobleaching, respectively.

For quantification of the colocalization, Pearson’s correlation coefficients were calculated with an ImageJ plugin, Colocalization Test (Wright Cell Image Facility, Canada).

### Short-circuit Current Measurements

Short-circuit current (I_SC_) measurements were performed in a six-channel Easy-Mount chamber system (Physiologic Instruments, San Diego, CA) that accepts Snapwell filters (Corning Costar, Acton, MA; 3407). I_SC_ was measured with a VCCMC6 multichannel voltage-current clamp amplifier (Physiologic Instruments) in the voltage-clamp mode. Data were acquired on an 1.71-GHz PC running Windows XP (Microsoft, Redmond, WA) and equipped with DI-720 (DATAQ Instruments, Akron, OH), with Acquire and Analyze version 2.3.159 (Physiologic Instruments) software. Cells were cultured to confluence on Snapwell filters before measurement. The cell monolayers were bathed on both sides with solution containing 115 mM NaCl, 25 mM sodium gluconate, 5 mM potassium gluconate, 1.2 mM MgCl_2_, 1.2 mM CaCl_2_, 10 mM D-glucose and 10 mM HEPES (pH 7.4 with NaOH). The mucosal side was replaced with a low Cl^−^ solution containing 139 mM sodium gluconate, 1.2 mM NaCl, 5 mM potassium gluconate, 1.2 mM MgCl_2_, 1.2 mM CaCl_2_, 10 mM D-glucose, and 10 mM HEPES (pH 7.4 with NaOH). The solution was constantly circulated, maintained at 37°C, and bubbled gently with air. Amiloride (20 µM) was added to the mucosal solution, and after stabilization, forskolin (10 µM) was added to the serosal chamber, followed by the CFTR channel inhibitor CFTRinh-172 (5 µM).

### Statistical Analysis

All quantitative results are given as averages ± standard error (SE). Statistical significance was determined using Student’s *t*-test. Values of p<0.05 were considered significant.

## Results

### CAL Binds to CFTR at the Golgi

**Figure 1:**
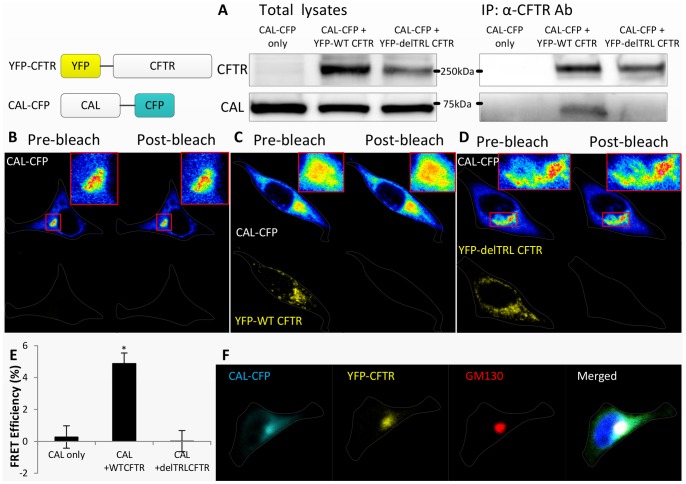
CAL-CFP binds YFP-CFTR in vivo, and CAL and CFTR are colocalized in the Golgi in HeLa cells. The N terminus of CFTR was tagged with YFP, and CFP was tagged to the C terminus of CAL was tagged with CFP. In HeLa cells, CAL-CFP was transiently expressed alone (A,B), with YFP-wildtype CFTR (A,C), or with YFP-delTRL CFTR (A,D). (A) Left panel: CAL-CFP, YFP-wildtype CFTR, and YFP-delTRL CFTR from total lysates were immunoblotted with anti-GFP antibody. Right panel: The cell lysates were immunoprecipitated with anti-CFTR antibody. (B-D) The left columns are the images before photobleaching with a 514-nm laser, and the right columns are the images after photobleaching. CFP and YFP are pseudocolored with a 16-bit lookup table (provided with ImageJ) and yellow, respectively. E shows the statistical analysis of the FRET efficiency obtained from the images. Results are means ±SE. (n = 11–13) *p<0.001. (F) YFP-CFTR and CAL-CFP were colocalized with GM130. HeLa cells that had been transfected with YFP-CFTR and CAL-CFP were immunostained with anti-GM130 antibody. The right-most panel shows the merged image of CAL-CFP, YFP-CFTR, GM130, and DAPI. (B–D, F). Cell outlines are drawn with gray lines.

To find out how CAL regulates CFTR trafficking, we investigated the interaction between CAL and CFTR by using an acceptor photobleaching FRET assay. For the assay, expression vectors of CFP-tagged CAL and YFP-tagged CFTR were constructed. As a first step, the binding of CAL-CFP and YFP-CFTR was confirmed by coimmunoprecipitation in HeLa cells (Fig. 1A). Next, CAL-CFP was transiently transfected into HeLa cells with YFP-CFTR or with YFP-ΔTRL CFTR, which does not bind CAL, and their FRET efficiency was then measured. The FRET results (Fig. 1B-E) confirmed the binding of YFP-CFTR and CAL-CFP, showing that the FRET efficiency of CAL-CFP and YFP-wildtype CFTR was significantly higher than that of the negative controls (CAL-CFP alone or with YFP-ΔTRL CFTR). These results demonstrate that CAL binds CFTR, but not ΔTRL CFTR, *in vivo* and that the detected FRET is specific for the binding of CAL to CFTR.

**Figure 2:**
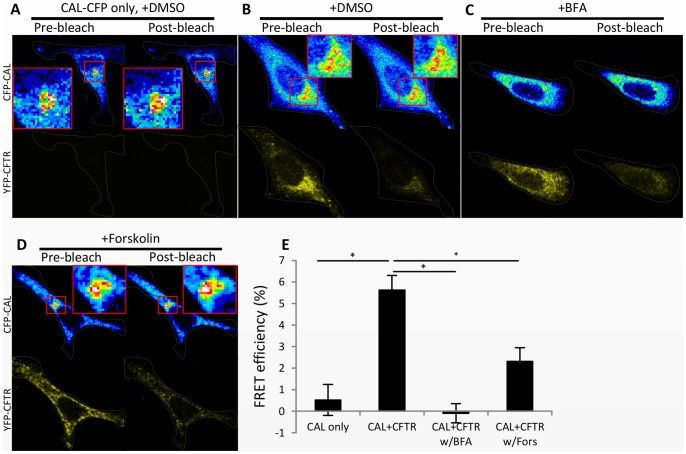
Disrupting the Golgi structure with brefeldin A and increasing the cell-surface expression of CFTR with forskolin diminish the FRET of CAL and CFTR. HeLa cells were transfected with CAL-CFP (0.5 µg) only (A) or with CAL-CFP (0.5 µg) and YFP-CFTR (1.5 µg) (B–D). DMSO (A, B), 10 µg/ml brefeldin A (BFA) (C), or 100 µM forskolin (D) was added at 37°C for 30 minutes. Images before (left columns) and after (right columns) photobleaching with a 514-nm laser are shown. CFP and YFP are pseudocolored with a 16-bit lookup table and yellow, respectively. Cell outlines are drawn with gray lines. (E) is the statistical analysis of the FRET efficiency of CAL and CFTR. The FRET efficiency of CAL and CFTR was reduced when BFA or forskolin was added. Results are means ±SE. (n = 44–68) *p<0.001.

The cell compartment in which CAL binds CFTR was determined by immunostaining for GM130, a Golgi marker. CAL-CFP and YFP-CFTR were colocalized with GM130 (Fig. 1F). Their colocalization within the Golgi and their binding suggest that CAL-CFP binds to YFP-CFTR in the Golgi. To support this result, we examined the FRET between CAL-CFP and YFP-CFTR after treatment with brefeldin A (BFA), a chemical that disrupts Golgi structure. Following treatment of cells with 10 µg/ml BFA at 37°C for 30 minutes, CAL-CFP was expressed throughout the cells, and the FRET between CAL-CFP and YFP-CFTR was reduced markedly (Fig. 2C,E). Thus, the *in vivo* binding of CAL-CFP and YFP-CFTR appears to depend upon the Golgi being intact, again suggesting that the Golgi apparatus is required for the binding of CAL-CFP and YFP-CFTR.

**Figure 3:**
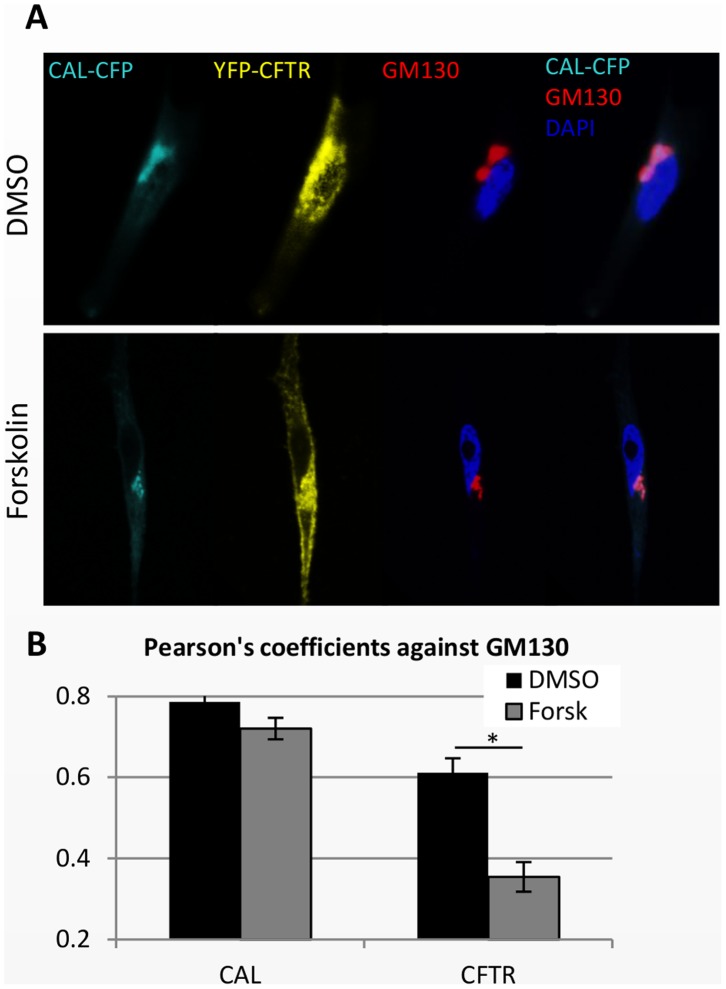
Forskolin treatment relocates CFTR, but not CAL, out of the Golgi in HeLa cells. HeLa cells were transfected with CAL-CFP (0.5 µg) and YFP-CFTR (1.5 µg). GM130 was immunostained with anti-GM130 antibody, followed by secondary antibody conjugated with Alexa-647. DMSO (A, upper panel) or 100 µM forskolin (A, lower panel) was added at 37°C for 30 minutes. (B) The graph shows Pearson’s correlation coefficients of GM130 and CAL or of GM130 and CFTR. The coefficients were calculated using an ImageJ plugin, the Colocalization Test. Pearson’s correlation coefficients of GM130 and CAL-CFP were not altered in the presence of forskolin, but those of GM130 and YFP-CFTR were reduced by forskolin treatment. Results are means ±SE. (n = 6–9) *p<0.01.

In order to examine more closely the trafficking of CAL and CFTR after forskolin treatment, we cotransfected 1.5 µg of YFP-CFTR and 0.5 µg of CAL-CFP expression plasmids into HeLa cells. After a 24-hour incubation, we treated the cells with 100 µM forskolin at 37°C for 30 minutes and immunostained the cells with an anti-GM130 antibody. We then used a plugin of ImageJ to calculate Pearson’s correlation coefficients to determine the quantitative colocalization of GM130 with CAL-CFP and YFP-CFTR. Interestingly, forskolin did not change the Pearson’s correlation coefficient between GM130 and CAL-CFP but reduced the coefficient between GM130 and YFP-CFTR (Fig. 3). Thus, following forskolin treatment, less CFTR was localized to the Golgi. Surprisingly, forskolin did not change the localization of CAL (Fig. 3), suggesting that the binding of CAL to CFTR occurs in the Golgi as shown above, but CAL does not move with CFTR out of the Golgi.

**Figure 4:**
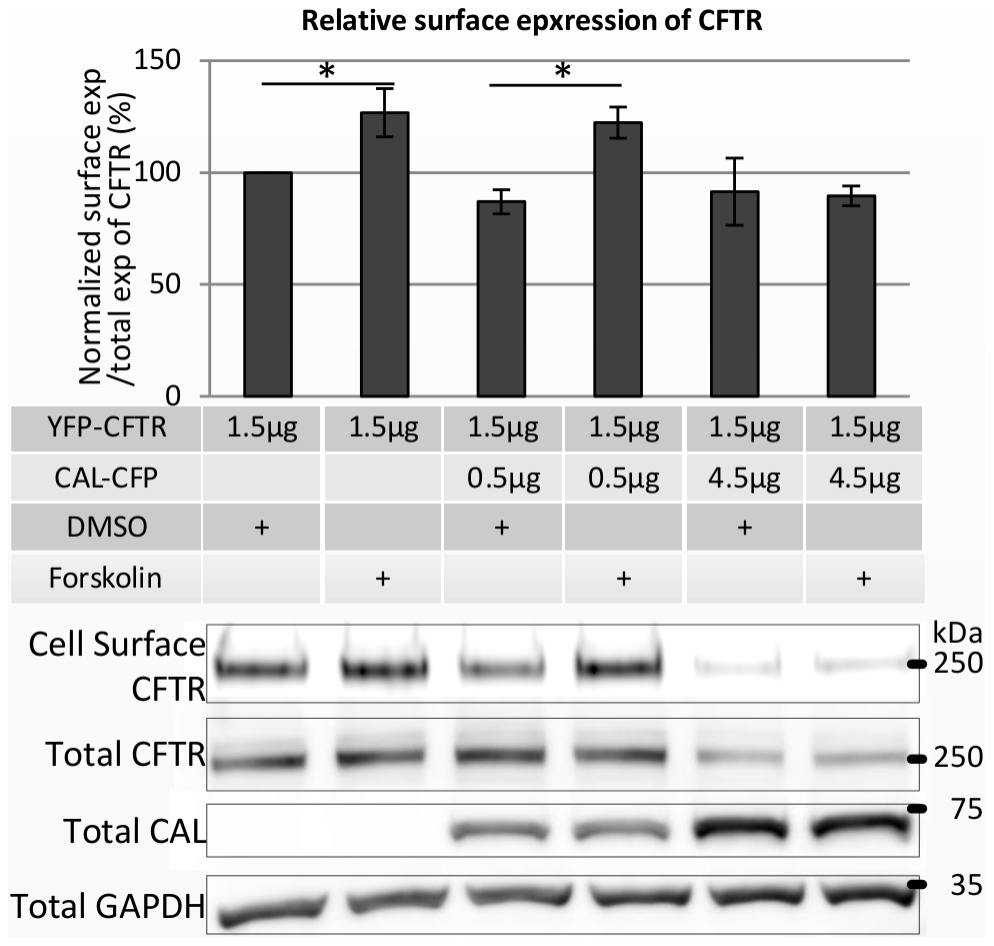
Forskolin fails to increase the cell-surface expression of CFTR with highly overexpressed CAL. HeLa cells were transfected with YFP-CFTR and CAL-CFP as indicated in the table. At 48 hours after transfection, cells were treated with DMSO or 100 µM forskolin at 37°C for 30 minutes. CFTR at the cell surface was then biotinylated. Cell surface-biotinylated CFTR, total CFTR, and total CAL were immunoblotted with anti-GFP antibody, and GAPDH was immunoblotted with anti-GAPDH antibody. The graph shows the ratio of the cell surface-biontinylated CFTR expression level to the total CFTR expression level, normalized by the ratio of the YFP-CFTR-alone samples with the DMSO treatment (%). When CAL was not overexpressed or 0.5 µg of CAL was transfected, forskolin increased the cell-surface expression of CFTR. However, when 4.5 µg of CAL was transfected, forskolin did not increase the cell-surface expression of CFTR. The total expression of CFTR was not altered by the forskolin treatment in all cases. Results are means ±SE. (n = 4) *p<0.05.

To verify that CFTR and CAL were not moving together to the plasma membrane in response to forskolin treatment, we examined the interaction between CFTR and CAL by the acceptor photobleaching FRET assay, utilizing the experimental conditions described above. More CFTR was present at the cell surface after forskolin treatment (Fig. 2D, Fig. 3, and CFTR-only sample results of Fig. 4), whereas CAL-CFP was still located in the cytosol (Fig. 2D and Fig. 3). Moreover, the treatment of forskolin reduced the FRET of CAL-CFP and YFP-CFTR (Fig. 2D, E), suggesting that forskolin reduces the association of CAL-CFP with YFP-CFTR and enhances CFTR movement to the cell surface, while CAL remains in the Golgi.

### Overexpression of CAL Inhibits the Forskolin-induced Promotion of CFTR Trafficking to the Cell Surface

To study the effect of CAL on the cell-surface expression of CFTR, we varied the doses of the CAL-CFP *versus* YFP-CFTR cDNA cotransfected into HeLa cells. We also performed a cell-surface biotinylation assay to measure the forskolin-induced changes in the cell-surface expression of CFTR. Fig. 4 shows that when YFP-CFTR was expressed alone, the 30-minute treatment with forskolin enhanced the CFTR expression at the cell surface, as previously shown [33], [34]. When the ratio of the transfected amount of CAL-CFP cDNA to YFP-CFTR cDNA was 1∶3, the same ratio we used in both the FRET assay and cell imaging studies, forskolin still increased the cell-surface expression of CFTR. However, when we transfected three times more CAL-CFP than YFP-CFTR cDNA (3∶1), forskolin no longer increased the surface expression of CFTR (Fig. 4). In all cases, forskolin did not change the total expression of CFTR (Fig. 4). Therefore, changes in CAL expression levels regulate CFTR’s progress to the cell surface.

### Active TC10 Alters CAL-CFTR Binding and Increases the Steady-state Levels of CFTR at the Cell Surface, Even with Highly Overexpressed CAL

**Figure 5:**
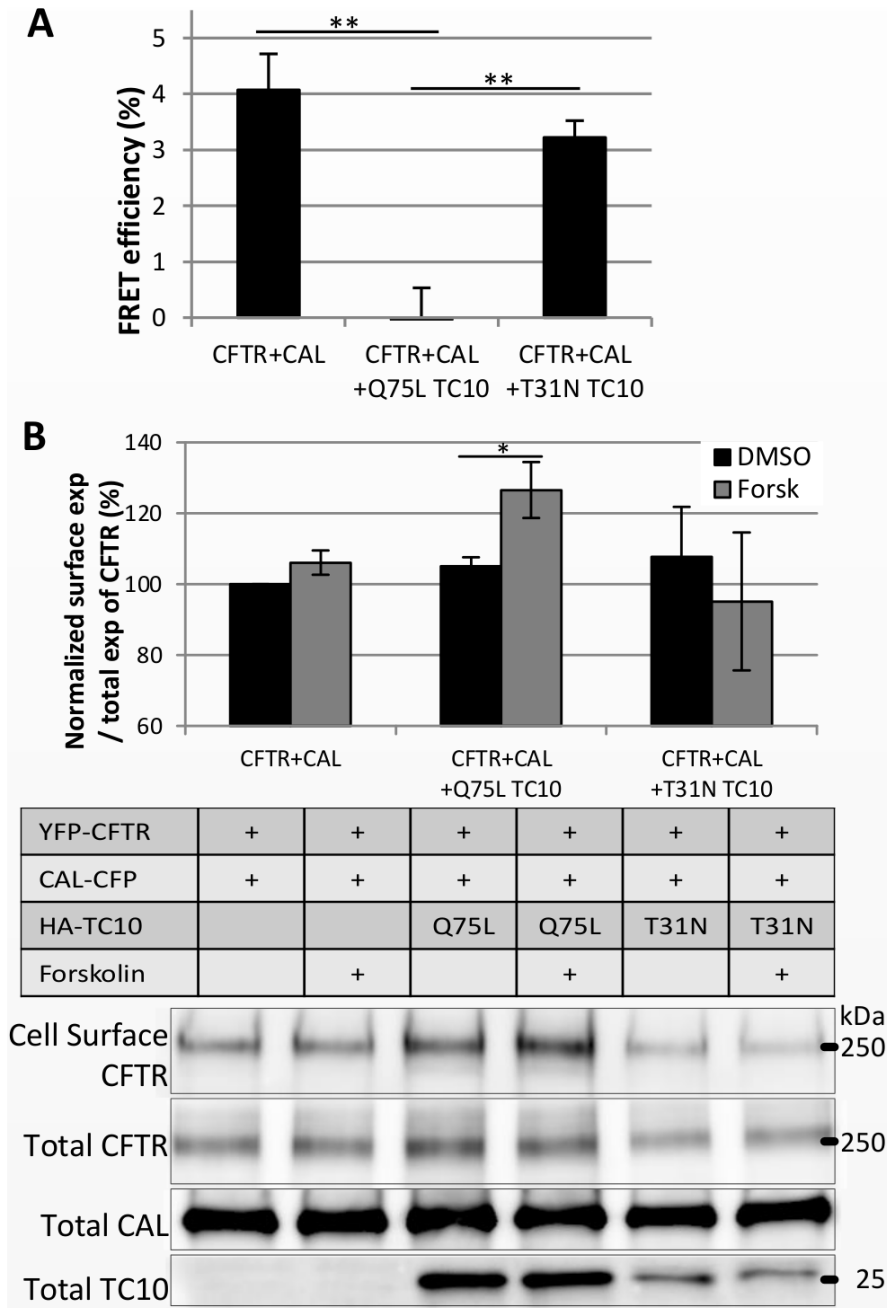
Active TC10 enhances CFTR expression at the cell surface, and this effect is impeded by highly overexpressed CAL HeLa cells were transfected with 1.5 µg of YFP-CFTR plus 0.5 µg (A) or 1.5 µg (B) of CAL-CFP expression vector without HA-TC10, with HA-Q75L TC10, or with HA-T31N TC10, as indicated. (A) The FRET efficiency of CAL and CFTR was reduced by a constitutively active mutant of TC10 (Q75L TC10) but not by a dominant-negative mutant, T31N TC10. (B) HeLa cells were treated with DMSO or 100 µM forskolin at 37°C for 30 minutes as described in the table. CFTR at the cell surface was biotinylated. The graph shows the ratio of cell surface-biontinylated CFTR to total CFTR, normalized by the ratio of the samples with YFP-CFTR and CAL-CFP treated with DMSO (%). Results are means ±SE. (In A, n = 7–13; B, n = 4) *p<0.05 and **p<0.005.

Previously, we showed that the constitutively active form of TC10 increases the surface expression of CFTR by binding to CAL [13]. We were curious to see whether active TC10 would also alter the CAL-CFTR interaction. The FRET assay showed that a constitutively active TC10 mutant, Q75L TC10, which remains in the GTP-bound form, does indeed reduce the FRET between CAL-CFP and YFP-CFTR. Importantly, the dominant-negative mutant T31N TC10, which remains in the GDP form, did not affect the FRET (Fig. 5A). These results indicate that active TC10 does indeed alter the binding of CFTR and CAL.

To test this possibility further, we cotransfected 1.5 µg of CAL-CFP cDNA with 1.5 µg of YFP-CFTR cDNA. This ratio inhibited the effects of forskolin on CFTR surface expression (Fig. 5B), as in Fig. 4. However, when the same amounts of CAL-CFP and YFP-CFTR cDNAs were cotransfected along with the active form of TC10 (Q75L TC10), forskolin was able to increase the surface expression of CFTR. Note that co-transfection of the dominant-negative form of TC10, T31N TC10, did not counter the effects of CAL overexpression, and in that case, forskolin did not increase CFTR surface expression (Fig. 5B). These data suggest that active TC10 enhances CFTR expression at the cell surface, even when CAL is overexpressed.

### IGF-1 increases the Steady State Levels of CFTR via TC10 and CAL

**Figure 6:**
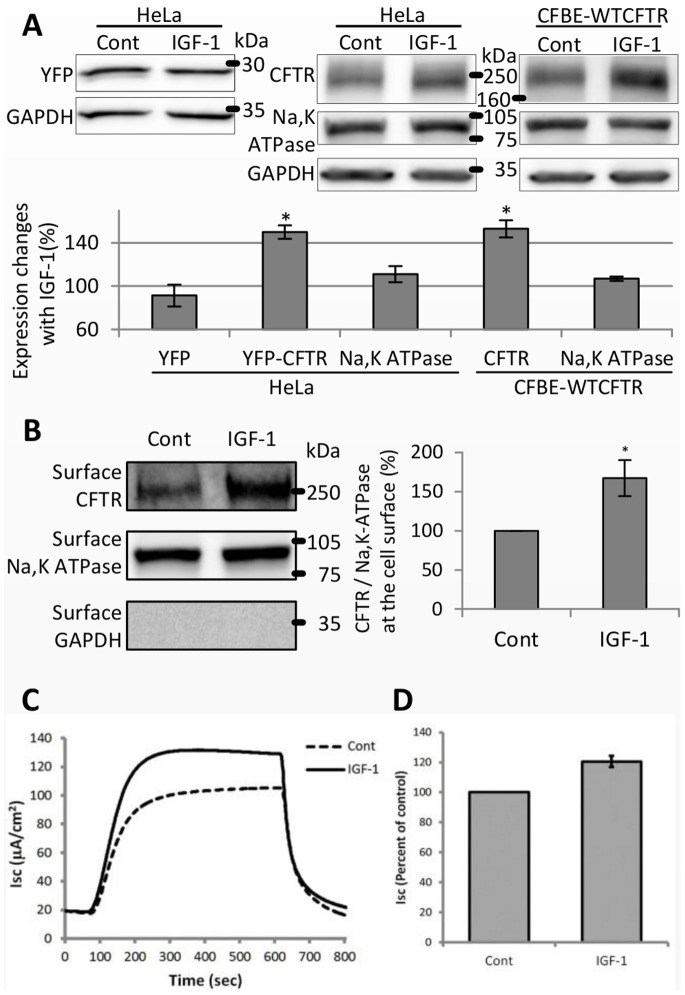
IGF-1 increases the protein expression level of CFTR and increases CFTR-mediated chloride transport in bronchial epithelial cells. (A) IGF-1 specifically increases the expression level of CFTR. HeLa cells were transfected with YFP or YFP-CFTR and incubated for one day, or confluent CFBE-WTCFTR cells were used. The graph in the right lower panel shows the expression of YFP, YFP-CFTR, CFTR, or Na,K-ATPase normalized by the GAPDH expression and normalized again by the corresponding control samples (%). (B) CFTR expression at the cell surface was increased by IGF-1 in HeLa cells. Proteins at the cell surface were cell surface-biotinylated. The graph in the right panel shows the expression of YFP-CFTR at the cell surface normalized by the Na,K-ATPase expression at the cell surface and normalized again by the corresponding control samples (%). (A,B) Cells were starved for 24 hours and then treated with 0.1 µg/ml IGF-1 for the next 24 hours. Results are means ±SE. (n = 3) (C) CFBE-WTCFTR cells grown on permeable supports were starved for 24 hours, then treated with 0.1 µg/ml IGF-1 for 24 hours. Cells were then mounted on the Ussing chamber setup, where short-circuit currents were measured as described in Materials and Methods. In the graph, 10 µM forskolin was added at the first arrow, and 5 µM CFTRinh-172 was added at the second arrow. (D) Ratio of I_SC_ between cells treated with 0.1 µg/ml IGF-1 (IGF-1) and untreated cells (Cont). Error bars represent SE. (n = 5). *p<0.05 and **p<0.005.

To determine whether the effects of TC10 are physiological, we utilized IGF-1, which is known to activate TC10 in neurons [21]. HeLa cells were transfected with YFP or YFP-CFTR, and after a 24-hour serum starvation, 0.1 µg/ml IGF-1 was applied for an additional 24 hours. IGF-1 increased the protein expression of YFP-CFTR but not of YFP alone (Fig. 6A); moreover, IGF-1 increased the CFTR expression level at the cell surface in HeLa cells (Fig. 6B). Because the two vector constructs share the same CMV promoter in the same backbone vector, the effects of IGF-1 on YFP-CFTR must be the result of post-transcriptional regulation. Furthermore, our results support the specificity of IGF-1′s effects on CFTR, in that IGF-1 treatment of HeLa cells did not change the protein expression of an endogenous plasma membrane protein, Na,K-ATPase (Fig. 6A).

To determine whether this regulation occurs in bronchial epithelial cells from cystic fibrosis patients (CFBE41o-, referred to as CFBE), we treated CFBE-WTCFTR cells (CFBE cells stably transfected with CFTR) with IGF-1. The effect was identical to that observed in the HeLa cells; IGF-1 increased the protein expression of CFTR but not of Na,K-ATPase (Fig. 6A). These data demonstrate that the effects of IGF-1 are not general, but specific for CFTR, and perhaps other proteins that share the same IGF-1-specific trafficking pathway.

To assess the function of CFTR in CFBE-WTCFTR cells, we measured CFTR-mediated Cl^−^ transport activity in IGF-1-treated cells. CFBE-WTCFTR cells grown on permeable supports were serum-starved for 24 hours, then treated with 0.1 µg/ml IGF-1 for 24 hours. High-resistance epithelial layers of CFBE-WTCFTR cells (whose average resistance was >1,200 Ω•cm^2^ for both control and IGF-1-treated cells) were mounted in Ussing chambers to measure the short-circuit current (I_SC_). As shown in Fig. 6C and D, the forskolin-activated I_SC_ in IGF-1 treated cells was significantly higher than the control cells. I_SC_ was completely inhibited by the CFTR inhibitor CFTRinh-172, showing that the currents were indeed CFTR-dependent.

**Figure 7:**
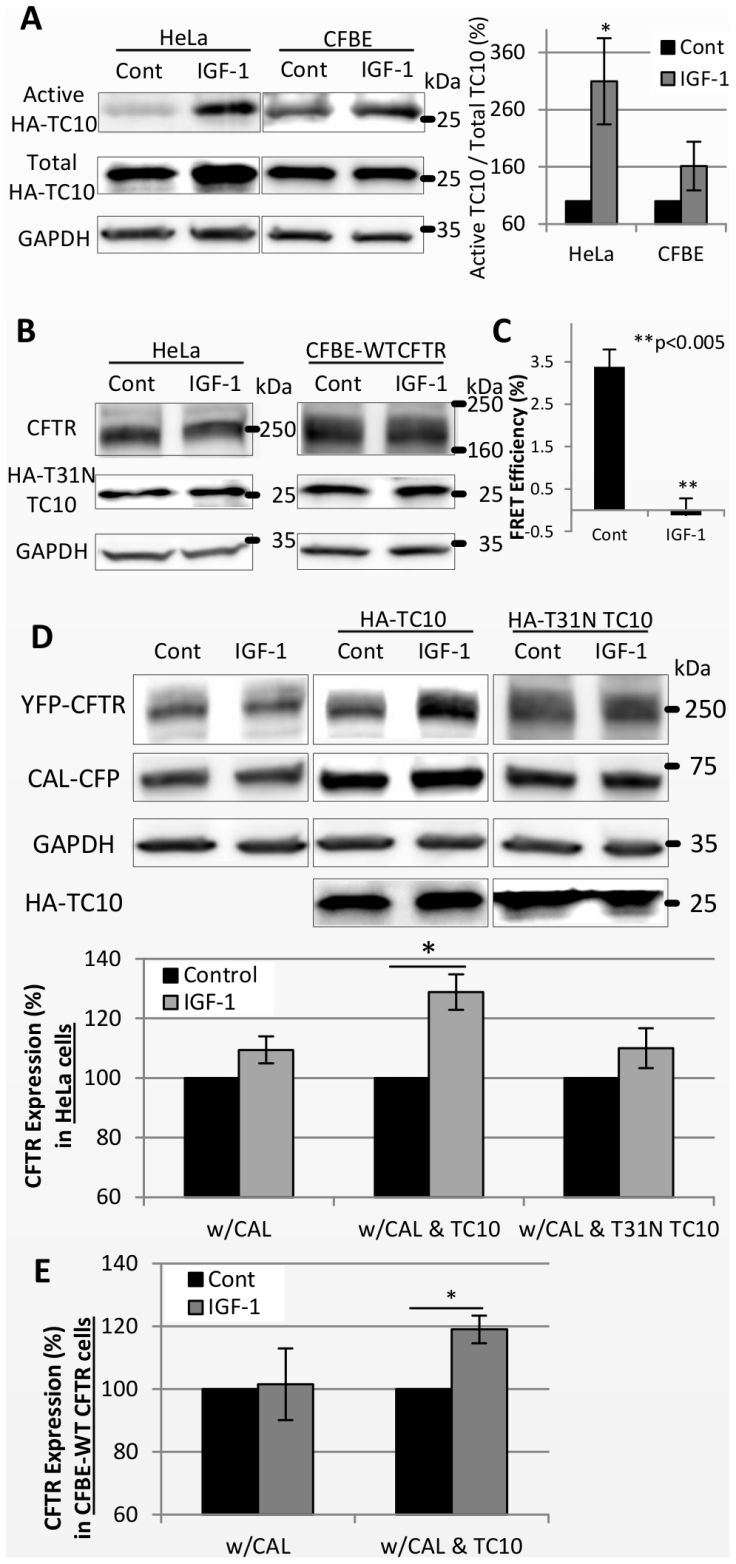
TC10 and CAL are involved in the IGF-1-mediated increase in CFTR expression level. (A) IGF-1 activates TC10. After HeLa cells or CFBE cells were transfected with HA-TC10, active TC10 was pulled down from the cell lysates with GST-PAK1 PBD peptide-conjugated beads. Total TC10 and GAPDH are from total cell lysates. The graph shows the amount of active TC10 normalized by the amount of total TC10 expression (%). (B) Dominant-negative TC10 (T31N TC10) inhibited the effects of IGF-1, increasing the CFTR expression. HeLa cells were cotransfected with YFP-CFTR and HA-T31N TC10, or CFBE-WTCFTR cells were transfected with HA-T31N TC10. Shown are representative images. (C) The FRET efficiency of CAL and CFTR was reduced by IGF-1. HeLa cells transfected with CAL and CFTR were starved for 4 hours and treated with 0.1 µg/ml IGF-1 for 30 minutes. (D,E) The CAL overexpression inhibited effects of IGF-1 increasing the CFTR expression, but the cotransfection with wildtype TC10 recovered the effects of IGF-1 on the CFTR expression in HeLa cells (D) and CFBE-WT CFTR cells (E). HeLa cells were transfected with YFP-CFTR and CAL-CFP without or with TC10 or T31N TC10. CFBE-WT CFTR cells were transfected with CAL-CFP without or with TC10. The CFTR expression level was normalized by the GAPDH expression level and normalized again by the corresponding control samples (%). (A,B,D,E) Cells were starved for 24 hours, and then 0.1 µg/ml IGF-1 was added for the next 24 hours. (A,C–E) Results are means ±SE. (n = 3) *p<0.05 and **p<0.005.

To determine whether IGF-1 activates TC10 in CFBE and HeLa cells, we used a peptide (PAK-1 PBD) that binds selectively to activated TC10 [31], [32]. Treatment with IGF-1 increased the level of activated TC10 in HeLa cells and in CFBE cells transfected with HA-tagged TC10 (Fig. 7A). When T31N TC10 was coexpressed with CFTR to suppress the endogenous activity of TC10, the IGF-1-induced increase of YFP-CFTR expression was abolished (Fig. 7B). Thus, activation of TC10 is required for IGF-1 to increase CFTR expression.

Given our observation that active TC10 reduced the FRET of CAL and CFTR, we reasoned that a similar mechanism might be involved in the IGF-1-mediated regulation of the steady-state levels of CFTR. Indeed, CAL-CFTR FRET was also reduced by IGF-1 (Fig. 7C). Therefore, we surmised that if IGF-1 increases the expression of CFTR by suppressing CAL, overexpression of CAL would reduce the effects of IGF-1. As expected, overexpression of CAL reduced the increase in CFTR expression induced by IGF-1 in HeLa cells (Fig. 7D). The effect of CAL overexpression could be reversed by the co-overexpression of TC10. In this case, IGF-1 still increased the steady-state levels of CFTR even when CAL was overexpressed (Fig. 7D). This result demonstrates that TC10, activated by IGF-1, can increase the steady-state levels of CFTR in a dominant fashion over CAL.

As shown in HeLa cells and also in CFBE-WTCFTR cells, IGF-1 increased CFTR protein expression via TC10 (Fig. 6A, Fig. 7B). This increase by IGF-1 was inhibited by CAL overexpression. However, CAL’s inhibition of IGF-1 was overcome by co-overexpression of TC10, again showing the dominance of TC10 in this process (Fig. 7E).

### IGF-1 Increases the Expression of Rescued ΔF508 CFTR, but not ΔF508 CFTR

**Figure 8:**
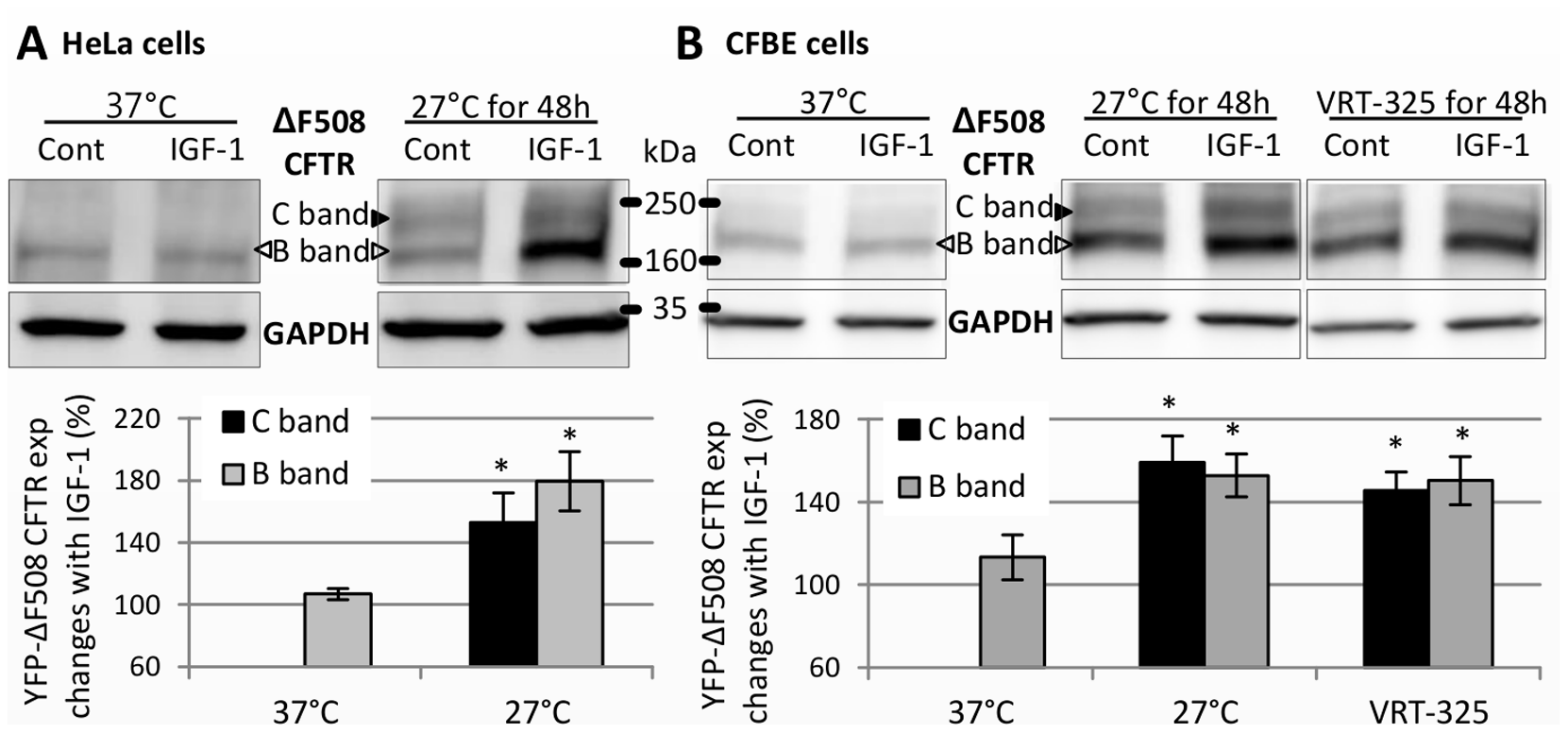
IGF-1 increases the expression level of rescued ΔF508 CFTR but not unrescued ΔF508 CFTR. HeLa cells (A) and CFBE cells (B) were transfected with YFP-ΔF508 CFTR, and after 1 day, plates of both cell lines were incubated at 37°C or at 27°C for 2 days; additional plates of CFBE cells were incubated with 5 µM VRT-325 at 37°C for 2 days. At the same time, 1 day after the transfection, these cells were serum-starved, and on next day, 0.1 µg/ml IGF-1 was added for 1 day. The expression level of ΔF508 CFTR in the cell lysates was assessed by western blotting. When unrescued (incubated at 37°C), ΔF508 CFTR showed one band (the B band), but when rescued (incubated at 27°C or with VRT-325 at 37°C), it showed two bands (B and C). IGF-1 did not increase the expression level of unrescued ΔF508 CFTR but did increase the expression level of rescued ΔF508 CFTR. The ΔF508 CFTR expression level was normalized by the GAPDH expression level, and the ΔF508 CFTR expression change as a result of IGF-1 treatment was then calculated by normalizing again with the non-treated control samples (%). Results are means ±SE. (n = 6 for HeLa at 27°C and CFBE at 37°C, n = 3 for all the others) *p<0.05.

ΔF508 CFTR is misfolded and resides primarily in the ER [4]. Most of the misfolded ΔF508 CFTR is degraded by ER-associated degradation and does not proceed to the Golgi. However, incubating cells at the low temperature [35] or treating with correctors [36]–[39] allows ΔF508 CFTR to proceed to the Golgi and the cell surface. Because ΔF508 CFTR is only core-glycosylated in the ER, it is detected by western blotting as a lower molecular weight B band, which resides primarily in ER; rescued ΔF508 CFTR, in contrast, is detected as a B band and a higher molecular weight C band, which is the Golgi-processed glycoform. We therefore transfected HeLa cells and CFBE cells with YFP-tagged ΔF508 CFTR and then rescued ΔF508 by incubating the cells at 27°C for 2 days [35] (Fig. 8). ΔF508 CFTR in CFBE cells was also rescued by incubating the cells with a corrector, VRT-325, at 37°C for 2 days [36] (Fig. 8B). IGF-1 did not increase the expression of ΔF508 CFTR; however, it did increase the expression of the C band of rescued ΔF508 CFTR as well as the B band of rescued ΔF508 CFTR (Fig. 8).

## Discussion

The question of how CAL regulates the trafficking of CFTR to the lysosome for degradation and, at the same time, to the cell surface remains unanswered. Where CAL molecules bind would be important information for understanding how CAL regulates CFTR. Our data have confirmed that CFTR and CAL are colocalized to the Golgi. We have also developed an acceptor photobleaching FRET assay to assess the binding of CAL and CFTR in the Golgi. Initially, we obtained FRET between CFTR labeled on the N-terminus with YFP and CAL labeled on the C-terminus with CFP and confirmed that the FRET assay showed the *in vivo* binding of CAL and CFTR. We then observed that when the normal structure of the Golgi was chemically disrupted, FRET was reduced, implying that the binding of CAL and CFTR was also disrupted and that the Golgi is required for their binding. When a decreased amount of CFTR was found in the Golgi after treatment with forskolin, which promotes the movement of CFTR from the Golgi to the cell surface, FRET between CAL and CFTR was also reduced. This finding supports the hypothesis that CAL binds CFTR and regulates the trafficking of CFTR at the Golgi.

Active TC10 has been shown to enhance CFTR expression [13], and we have found that it promotes the trafficking of CFTR to the cell surface, even in the presence of highly overexpressed CAL, suggesting that the role of TC10 is dominant over CAL. One possible mechanism to explain these findings is that active TC10 disrupts the binding of CAL to CFTR by proceeding with CFTR to the plasma membrane and leaving CAL behind in the Golgi. Alternatively, TC10 could alter the conformation of the CFTR-CAL complex, thereby increasing the distance between the fluorescent tags on the N-terminus of CFTR and on CAL that bind to the C-terminus of CFTR. This alteration in conformation would also reduce the FRET of CAL and CFTR by active TC10, while CAL still binds CFTR. Indeed, our lab has shown in a previous report by coimmunoprecipitation that CAL and CFTR remain associated following active TC10 overexpression [13]. TC10 is known to bind to the N-terminal coiled-coil domain of CAL and not to the PDZ domain where CFTR binds [17]; thus, it is not surprising that CAL can still bind CFTR in the presence of TC10. TC10, by inducing conformational changes, might move the whole CAL-CFTR complex away from the Golgi toward the plasma membrane, since our previous report showed that active TC10 relocates CAL toward the plasma membrane in COS7 cells [13]. At or near the plasma membrane, CFTR would competitively bind to NHERF, which has a higher affinity for the PDZ domain of CFTR than does CAL [40], and thereby enhance the CFTR expression at the plasma membrane by stabilizing CFTR [41].

TC10 has two known mechanisms for regulating membrane trafficking: It recruits excocyst complex proteins (e.g., Exo70, Sec6, and Sec8) to GLUT4 vesicles in adipocytes and to plasmalemmal precursor vesicles in neurons [21]–[23]. These complexes are involved in the exocytosis of membrane vesicles. Secondly, TC10 uses PI3K-dependent signaling by increasing phosphatidylinositol 3-phosphate (PI3P) [42]–[44]. The increased PI3P level induces exocytosis of membrane vesicles and raises the intriguing possibility that when present together in cells, GLUT4 and CFTR may traffic together to the plasma membrane in the same TC10 activated pathway. The current study proposes a novel, third mechanism for TC10 regulation whereby active TC10 alters the binding of a cargo molecule, CFTR, to an adaptor protein, CAL, and thereby enhances the trafficking of the cargo (i.e., CFTR) to the plasma membrane.

TC10 is located downstream of the IGF-1 receptor cascade [21]–[23]. We show here that IGF-1 activates TC10 and increases CFTR expression in CFBE and HeLa cells, but does not increase CFTR expression in the presence of the dominant-negative TC10. Furthermore, we have shown that IGF-1 increases CFTR expression by activating TC10. As we saw in the case of constitutively active TC10, IGF-1 alters the CFTR-CAL interaction and enhances CFTR expression.

In bronchial epithelial cells, adequate expression of the CFTR protein is required to prevent bacterial infection, which is the most common CF symptom [45], [46]. The reports that bronchial epithelial cells express the IGF-1 receptor [47] and react to IGF-1 [48], [49] suggest that these cells may have IGF-1 signaling. Our results also indicate that that IGF-1 selectively increases CFTR protein expression and CFTR-mediated chloride transport in a cell line model, CFBE cells (the bronchial epithelial cell line from cystic fibrosis patients). Therefore, our results raise the possibility that IGF-1 treatment could increase CFTR expression in vivo.

Serum IGF-1 levels vary physiologically and pathologically in individuals in response to aging, caloric uptake, injury, and other conditions, and they regulate IGF-1 signaling, causing mitogenic effects such as cell proliferation, tissue regeneration, and body growth [50]–[53]. According to our present results, changes in the level of IGF-1 would be expected to alter CFTR protein expression. CAL also binds other plasma membrane proteins such as the β1-adrenergic receptor, ClC-3B, somatostatin receptor subtype 5, and cadherin 23, and enhances their lysosomal degradation [54]–[57]. If TC10 also regulates the CAL-dependent degradation of these proteins, IGF-1 could also post-translationally regulate the expression levels of these plasma membrane proteins. IGF-1 levels are low in the serum of CF patients and the CF pig model [26], [27]; therefore, IGF-1 has been studied and used in a clinical trial to reverse growth retardation in CF patients [25], [58].

ΔF508 CFTR, the most common CF mutation, is degraded by ER-associated degradation and does not reach the Golgi or plasma membrane [59]. Our results show that IGF-1 does not change the expression level of ΔF508 CFTR, consistent with our proposal that IGF-1 alters the binding of CAL to CFTR at the Golgi, since almost all of ΔF508 CFTR is sequestered in the ER and does not proceed to the Golgi [60]. However, IGF-1 does increase the expression of rescued ΔF508 CFTR, because when ΔF508 CFTR is rescued by correctors or low temperature, it moves out of the ER to the Golgi and the cell surface [35], [38], [61]. Interestingly, IGF-1 does not increase the intensity of un-rescued ΔF508 CFTR band B, whose N-glycosylation is initiated in the ER but not finalized in the Golgi; however, it does increase its intensity when ΔF508 CFTR is corrected by low temperature or VRT-325. These data suggest that the rescued immature B band of ΔF508 CFTR may in some instances exit the ER through an unconventional trafficking pathway, perhaps enhanced by TC10 acting through IGF-1, and reach the plasma membrane in an immature form [62]. However, more work is needed to establish this effect conclusively. Correctors have been developed and are currently in clinical trials [37], [38]. Thus, it is possible that an additive treatment combining IGF-1 with a corrector would be more effective for cystic fibrosis than a corrector alone.
